# Correspondence of Prof. W. H. Byford

**Published:** 1860-07

**Authors:** Robert Robson

**Affiliations:** New Harmony


### CORRESPONDENCE OF PROF. W. H. BYFORD.

MY DEAR SIR:

How comes on your new College and *Medical Examiner?* Do me the favor to forward the *Examiner* to my address, and I will with pleasure remit you the amount, and send you an occasional article for its columns. I feel some anxiety to know something more of Chicago, and her institutions, and particularly of the professional career of my friend Dr. Davis and yourself. I have not the least doubt of the ultimate success of Lind College. It is my opinion that you will have a good winter class. Students will have all the advantages in your Institution in Chicago which are accessible in any other large city. From personal knowledge, I am satisfied they will have the opportunity to see as much practice both in Medicine and Surgery as anywhere else; this I deem all important to the student previous to entering upon the duties of the profession.

In reference to your former locality in Indiana, I would observe that but few changes of interest have transpired. Our summer fevers are not perhaps of such frequent occurrence in proportion to our present population as formerly, or in the fall season of so malignant a type as at the time when you resided among us. It is true that time and fashion has imposed upon us some change in the treatment of disease, but why should we expect to be exempt from what appears to be the common lot of all? Remedial agents and modes of practice, have all of them had their periods of celebrity, decline, and revival; and if we will take the trouble to examine the antecedents of the past, we will find, alas! that first one system and then the other have been in use before, and have in time sunk into neglect; such seems to be the fate of emetics here, as a remedial agent in the various grades of bilious fevers; and also venesection in pneumonia has fallen into very general disuse in this section. I know of no reason for the abandonment of the proper use of these therapeutic remedies. We certainly have no new pathological forms of summer fevers now, that has not existed to a greater or less extent ever since I have known the locality. I have, for upwards of thirty years, very generally used emetics in the commencement of bilious fevers, and certainly have not the slightest cause to regret their use. You are aware, Sir, from practical experience, that our summer fevers consist, with the exception of the typhoid form, very generally of intermittents and remittents, generally of a high grade of action. The more violent forms are characterized by a severe pain in the forehead, sometimes delirium, pains in the lumbar and epigastric; often in the left, and sometimes in the right hypogastric regions; hurried respiration, flushed face, aching in the bones, dry and hot skin, intense thirst, tongue covered with a thick yellowish white coat, and sometimes with a yellowness of the eyes and skin, urine high colored, and the bowels generally constipated; with a full and strong pulse, nausea and frequent vomiting of bilious matter. In the above form of fever, emetics produce effects of a kind, and with a speed which no purgative alone can equal. You will recollect the unusual sickly year of 1833 or '34, when almost every family were more or less affected by the above form of fever; some hundreds of such cases were in my hands during that season, and emetics were very generally used in the onset, and with the exception of my distinguished patient, Professor Say, I had not a single death from fever during the whole summer. I do not say this, Sir, from any feeling of egotism, but simply because it is true.

The occurence of those fevers at the same season of the year; their production from the same local causes, with some slight variations; their coincidence of general symptoms; and hitherto, the very successful mode of practice pursued, certainly points to a perseverance in the same curative plan.

What substitute have we to supply the place of emetics in such cases? Their influence goes far beyond the mere emptying of the morbid contents of the stomach and alimentary canal; the nausea which attends their operation, and the mechanical pressure of the diaphragm during the act of vomiting, brings the liver and whole portal circulation under their beneficial influence. The severe attendant headache in the frontal portion of the brain, and the congestion, which so often attends the hypogastric region, is not only removed by emetics, but the Ductus Communis Choledochus is relaxed, and the passage of the bile flows freely into the duodenum, and results very often in evacuating the bowels, rendering at the time more medicine unnecessary, while the relaxed and moist surface tends materially to lessen the febrile action.

I am aware that however specious the modus operandi of emetics may appear, that many timid practitioners reject their use as inadmissible, in consequence of the apparent prostration of the energies of the system which they produce; but this is temporary in character, and fully compensated for in a few hours by the improved condition of the patient.

It appears to me that if we ever do attain to anything approaching a correct mode of practice, it must be by the incessant accumulation of facts. I have no objection to theories, or know of no impropriety in the application of theories to facts, if made in the true spirit of inquiry, by analizing and comparing fact after fact, and drawing from them such conclusions as will apply to other facts of a similar kind. To investigate disease is to observe facts, and to note their indissoluble connection between cause and effect. This is the only theory which I can recognize—the observation of facts, and the points in which the causes which produce them agree, and making in general terms their features of resemblance, and thus forming a principle to guide us in the cure of disease.

It is upon the accurate discrimination of the physician amidst the various shades of constitution and disease, and a reference of each symptom to its proper antecedent, that he is enabled to achieve that which distinguish him from the common herd of the profession.

On the subject of Venesection in Pneumonia I have not much to say. However beneficial it may be in the early stage, where there is a full and strong pulse, it certainly is not admissible in the more advanced stage. Physicians in this section are seldom called upon in a country practice to visit the patient in time to use the lancet. Much harm has been done by indiscriminate bleeding, and if extremes must be adopted by a certain class of practitioners, I am of the opinion that the community will be much the gainer by their not bleeding at all.

I had almost forgot to mention that we have had numerous accessions to the faculty of Southern Indiana since you left. Many are gentlemen, and promise to be an honor to the profession, while others in this section, as elsewhere, are merely illiterate tyros in practice, and *practically* discard all knowledge of Materia Medica, Pharmacy, etc. With some exceptions, the only article deemed necessary to their success is Hydrag. Submur.; and while I appreciate the proper use of this article to the various purposes of disease, I regret to be under the necessity of making this statement. No remedy has been more successfully or extensively used, or in a greater number of cases, yet it is nevertheless a fact, that no article is so shamefully abused; a resort to its use is too often *the refuge of ignorance and incompetence.* Such men use it in all stages of febrile action. They employ it almost, if not exclusively, in greatly excited states of the heart and arteries. Should there be local disease in connection with the fever, it is given in all its stages, and often without regard to the propriety of previous evacuations. It is given in minute doses. It is used in order that it may exert a specific stimulant influence independent of its known properties. It is employed to destroy the action of fevers by its own mercurial action on the system.

In early life I frequently witnessed the failure of large doses of calomel to produce evacuations, when I have found in nearly similar cases, and under nearly similar circumstances, a more mild, yet more active evacuant, answer every purpose, and accounted for it by the debility of the intestinal canal being increased by the stimulus of the mercury. I have known dose after dose to be given by some practitioners without producing a single passage, and in my opinion because the stomach and bowels were rendered insusceptible by the very means used to increase their peristaltic motion and empty their contents. When such men are doubtingly and despondingly sitting at the bed-side of a patient whose doubtful case calls forth their intense anxiety—their minds bewildered in uncertainty and and doubt, when their favorite remedy—this single club of Hercules—this lever and fulcrum of Archimides is about to disappoint their cherished hopes—how admirably convenient, they have but to push still more energetically the chloride; with this alone they feel themselves competent to slay the monster disease, and set at defiance hosts of opposition.

I was present at the National Medical Convention, held in Philadelphia in 1847, and felt proud of my profession; certain it was, that there never was a more noble or talented body of men assembled together than there was on that occasion, and from the sentiments there and then expressed by many distinguished physicians, I had hoped that something would have been accomplished ere this to elevate the standard of the medical profession. Till this is done you may expect to embrace in our ranks, men of every shade of intellect, if not some that scarcely possess any.

Some considerable time since I received from you a letter, soliciting from me contributions for the pages of the *Examiner*, and I do not recollect whether I replied to you or not, but be this as it may, I would say to you that for some time I have been more or less indisposed, and have not written so much as formerly, but will endeavor to become a better correspondent in future. I will send you two or three of my recent cases, which will, perhaps, be of some interest to the profession, and forward you enclosed for publication.

Please present my respects to Dr. Davis, and accept the same for yourself.

Respectfully Yours,

*New Harmony*, June 9th, 1860. ROBERT ROBSON, M. D.

